# Mouth rinsing with a carbohydrate solution attenuates exercise-induced decline in executive function

**DOI:** 10.1186/s12970-017-0200-0

**Published:** 2017-11-22

**Authors:** Kana Konishi, Tetsuya Kimura, Atsushi Yuhaku, Toshiyuki Kurihara, Masahiro Fujimoto, Takafumi Hamaoka, Kiyoshi Sanada

**Affiliations:** 1 Graduate School of Sport and Health Science, Ritsumeikan University, Kyoto, Shiga Japan; 2 Graduate School of Human Development and Environment, Kobe University, Kobe, Japan; 3 Faculty of Sport and Health Science, Ritsumeikan University, Kusatsu, Shiga Japan; 4 Department of Sports Medicine for Health Promotion, Tokyo Medical University, Tokyo, Japan

**Keywords:** Maltodextrin and mouth washing, Executive function, Stroop color and word test

## Abstract

**Background:**

A decline in executive function could have a negative influence on the control of actions in dynamic situations, such as sports activities. Mouth rinsing with a carbohydrate solution could serve as an effective treatment for preserving the executive function in exercise. The purpose of this study was to examine the effects of mouth rinsing with a carbohydrate solution on executive function after sustained moderately high-intensity exercise.

**Methods:**

Eight young healthy participants completed 65 min of running at 75% V̇O_2_max with two mouth-rinsing conditions: with a carbohydrate solution (CHO) or with water (CON). Executive function was assessed before and after exercise by using the incongruent task of the Stroop Color and Word Test. The levels of blood glucose; and plasma adrenocorticotropic hormone (ACTH), epinephrine, and norepinephrine (NE) were evaluated. A two-way repeated-measures ANOVA, with condition (CHO and CON) and time (pre-exercise and post-exercise) as factors, was used to examine the main and interaction effects on the outcome measures.

**Results:**

The reaction time in the incongruent condition of the Stroop test significantly increased after exercise in CON (pre-exercise 529 ± 45 ms vs. post-exercise 547 ± 60 ms, *P* = 0.029) but not in CHO (pre-exercise 531 ± 54 ms vs. post-exercise 522 ± 80 ms), which resulted in a significant interaction (condition × time) on the reaction time (*P* = 0.028). The increased reaction time in CON indicates a decline in the executive function, which was attenuated in CHO. Increases in plasma epinephrine and NE levels demonstrated a trend toward attenuation accompanying CHO (*P* < 0.085), which appeared to be associated with the preservation of executive function. The blood glucose concentration showed neither significant interactions nor main effects of condition.

**Conclusions:**

These findings indicate that mouth rinsing with a carbohydrate solution attenuated the decline in executive function induced by sustained moderately high-intensity exercise, and that such attenuation seems to be unrelated to carbohydrate metabolic pathway but rather attributed, in part, to the inhibition of the excessive release of stress hormones.

## Background

Executive function, the cognitive process that modulates behavior by shifting and updating goal-directed strategies and inhibition of dominant responses, plays an important role in the control of action in situations that require to change and update goal-directed strategies in a dynamic environment [1–4]. Some previous studies [5, 6] have shown that executive function was impaired during and after sustained (> 60 min) high-intensity exercise (>70% V̇O_2_max) [7, 8], which causes several changes in modulators of executive function, such as arousal or the levels of some brain neurotransmitters [9, 10]. Such exercise-induced impairment in executive function would have a negative influence on the control of action in dynamic situations that require sustained moderately high-intensity exercise such as sports activities. Therefore, it is necessary to investigate effective interventions that can help preserve executive function in such exercise.

The positive effects of mouth rinsing with a carbohydrate solution on executive function have recently received increased attention as an alternative to carbohydrate ingestion [11–13]. It has been reported that the ingestion of carbohydrate drinks enhances executive function in both resting and exercise conditions [5, 14–16]. However, carbohydrate ingestion might not be an optimal treatment form to preserve executive function in sustained high-intensity exercise, as it often induces gastrointestinal discomfort [17–19] and sustained high-intensity exercise induces deterioration in global brain glucose uptake [20]. Mouth rinsing with a carbohydrate solution, even without carbohydrate ingestion, has been reported to improve executive function in a resting condition [12, 13], suggesting that its effect is induced through non-energetic mechanisms. Mouth rinsing with a carbohydrate solution would attenuate a decline in executive function through the activation of reward pathways and/or vagus nerve. Previous studies demonstrated that carbohydrate mouth rinsing enhanced executive function [12] and exercise performance [21] because the taste of carbohydrate solution increased cortical response in motivational reward pathways. Another study suggested that the carbohydrates solution enhanced executive function via vagus nerve innervation [22]. As its favorable effect on executive function appears to be unaffected by exercise-induced deterioration in brain glucose uptake, and because it does not cause gastrointestinal discomfort, mouth rinsing with a carbohydrate solution could be a better form of intervention aimed at preserving executive function in sustained moderately high-intensity exercise. However, to our knowledge, no study has examined the effect of mouth rinsing with a carbohydrate solution on exercise-induced changes in executive function.

Therefore, the purpose of this study was to examine the effects of mouth rinsing with a carbohydrate solution on executive function in sustained moderately high-intensity exercise. The primary outcome of the present study was the reaction time associated with executive function via the incongruent condition of the Stroop Color and Word Test. The secondary outcomes were the response accuracy of the incongruent Stroop test and the subjective perception, as well as the blood levels of metabolites and hormones. We hypothesized that mouth rinsing with a carbohydrate solution would attenuate the exercise-induced decline in executive function in this condition.

## Methods

### Participants

Eight young healthy participants (four males and four females; mean age, 24.1 ± 1.8 years; body mass, 64.1 ± 4.2 kg; height, 170.6 ± 5.0 cm; and V̇O_2_max, 42.4 ± 8.3 ml⋅kg^−1^⋅min^−1^) participated in this study. None of the participants had a history of mental or somatic disorder or had engaged in regular endurance training for a minimum of 1 year. This study was performed in compliance with the Declaration of Helsinki. The study protocol was approved by the Ethics Review Board of Ritsumeikan University Biwako-Kusatsu Campus (BKC-IRB-2014-034). Each participant received an explanation of the purpose and potential risks of the study, as well as about the experimental procedure, and provided written informed consent for participation.

### Maximal graded exercise test

The participants underwent a maximal exercise test to determine their exercise intensity at least 5 days before the experiment. Oxygen uptake was measured with a breath-by-breath gas and volume analyzer (AE-310S; Minato, Osaka, Japan) during running on a treadmill (Life Fitness, Tokyo, Japan). The participants ran at a constant inclination of 1%, and the speed was increased by 0.9 km/h every 1 min from 6.0 km/h. Heart rate was recorded continuously during the exercise test by using a heart rate monitor (Polar RS800CX; Polar Electro Oy, Kempele, Finland). V̇O_2_max was considered to be valid when at least two of the following three criteria were met: a plateau in V̇O_2_ **,** defined as when V̇O_2_ at two different grades differs by less than 2.1 ml⋅kg^−1^⋅min^−1^ [23], a respiratory exchange ratio of ≥1.10, and attainment of at least 90% of the age-predicted maximal heart rate (220 – age) [bpm] [24].

### Experimental protocol

Each participant completed two exercise sessions with two different mouth-rinsing conditions during the exercise: either with a carbohydrate solution (CHO) (Maltodextrin; Body Plus International, Miyagi, Japan) or with water (CON). Participants were blinded to the mouth-rinsing condition. The exercise sessions with different mouth-rinsing conditions (i.e., CHO and CON) were performed at intervals of at least 5 days, with randomized order. Participants were familiarized with the experimental protocols prior to the exercise sessions. For female participants, the experiment was conducted during the follicular phase of the menstrual cycle.

With regards to the participants’ diet, they were supervised to ensure that all of them had the same diets on the days prior to the first and second experiments. On the day before each session, the participants were instructed to have dinner by 22:00 and not consume anything other than water after the meal. On the following morning, the participants visited the laboratory and had breakfast at 8:20. The provided breakfast was identical for all participants and was equivalent to 485 kcal (18.8 g protein, 10.1 g fat, and 75.6 g carbohydrate).

**Fig. 1:**
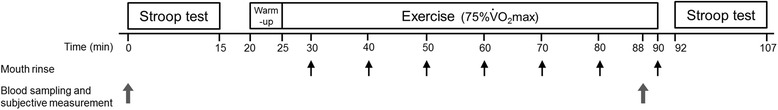
A schematic outline of the present study. Stroop test was administered before and after exercise (approximately 15 min for each test). Mouth rinsing was performed every 10 min from the start of exercise (indicated by small arrows). Immediately before each Stroop test, subjective perceptions were measured and a sample of blood was taken (indicated by large arrows)

A schematic outline of the present study is shown in Fig. 1. Each exercise session was started 3 h after the breakfast. The participants ingested water (5 mL/kg body weight) before the exercise [25]. Then, each participant ran for 5 min at a workload corresponding to 40%V̇O_2_max as a warm-up, which was followed by 65-min running at 75%V̇O_2_max on a treadmill at a constant inclination of 1%. The mean room temperature was 20 °C. Heart rate was recorded continuously during the exercise. Mouth rinsing was performed every 10 min from the start of exercise.

A cognitive function test was administered before exercise (pre-exercise) and after exercise (post-exercise: within 2 min after the exercise session). Immediately before each cognitive function test, subjective perceptions were assessed and a blood sample was taken.

### Mouth rinse solution

In CHO, a 6.4% maltodextrin (Maltodextrin; Body Plus International, Miyagi, Japan) (6% carbohydrate) solution was used. Maltodextrin is colorless and unsweetened when dissolved in water. The participants were instructed to rinse their mouths with a mouthful (approximately 25 ml) of the solution for 5 s before spitting into a bowl. The carbohydrate concentration and the volume of the rinsing solution were decided on the basis of previous studies [26, 27].

### Executive function test

The modified incongruent Stroop Color and Word Test [28] was administered to assess executive function of the participants. A computer screen that displays different words with different colors was placed 1 m in front of the participants at their eye level in the sitting position. A white fixation cross (+) on a black background appeared for 750 ms followed by word stimulus presentation with a duration adjusted for each participant and a blank black screen for 750 ms. For the incongruent condition, the words “BLUE,” “GREEN,” or “RED” in Japanese were displayed in a different ink color (e.g., BLUE printed in green ink). The congruent version of the Stroop Color and Word Test was used as control condition, in which the words “BLUE,” “GREEN,” or “RED” were displayed in the congruent ink color (e.g., BLUE printed in blue ink). The congruent task is known to measure mainly selective attention and concentration [29]. The participants were asked to identify the color of the displayed word by pressing the appropriate key on the keyboard, with the index, middle, and fourth fingers of their right hand (the dominant hand of all participants). They were then instructed to press the key in accordance with the color of the ink and ignore the semantic meaning as accurately and quickly as possible in the modified incongruent Stroop Color and Word Test. The test consisted of six blocks of 25 trials each, for a total of 150 trials. The trial conditions (congruent and incongruent) were set in a random order.

On the day before the experiment, all participants repetitively practiced reaching the level of >80% of response accuracy [30–32] and to reach a stable level of reaction time, to minimize the learning effects. The task difficulty was controlled by adjusting the stimulus duration from 650 to 1000 ms, so that each participant could maintain a > 80% response accuracy.

A software (E-Prime 2.0; Psychology Software Tools, Sharpsburg, PA, USA) was used to present the stimuli and measure the reaction time. The reaction time and response accuracy were averaged for each trial condition. Trials in which the reaction time was recorded to be <120 ms were excluded from the calculation.

### Blood analysis

An indwelling needle was inserted into a median cubital vein after breakfast and a 12-mL venous blood sample was taken at each time point. The glucose concentrations were measured by using an automated glucose analyzer (FreeStyle FreedomLite; Nipro, Osaka, Japan). The blood samples were each dispensed into a collection vessel for the analysis of adrenocorticotropic hormone (ACTH), epinephrine, and norepinephrine (NE). All samples were centrifuged at 3000 rpm for 15 min at 4°C. The levels of plasma ACTH, epinephrine, and NE were analyzed by means of reverse-phase isocratic high-performance liquid chromatography (HPLC) at a clinical laboratory (MEDIC, Yasu, Japan).

### Subjective measurements

The rating of perceived exertion (RPE) was measured with the Borg 15-point scale, which ranges from 6 (very, very light) to 20 (very, very heavy). Perceptual fatigue, arousal (from extremely high energy to extremely low energy), and pleasure were assessed by using a scale ranging from 1 (not at all) to 20 (maximal). Participants orally responded to these questionnaires before and during exercise.

### Statistical analyses

The outcome measures were the reaction time of the Stroop Color and Word Test, the subjective perception, and the blood levels of metabolites and hormones. Data are reported as mean (SD) unless otherwise noted. A two-way repeated-measures ANOVA, with condition (CHO and CON) and time (pre-exercise and post-exercise) as factors, was used to examine the main and interaction effects on the outcome measures. When significant interactions were found, Bonferroni post hoc test was performed to detect the sources of the significant differences. In all analyses, *P* < 0.05 was used to indicate statistical significance. Partial eta squared (${\eta}_p^2$) were reported as estimates of effect size. The data were analyzed with SPSS (version 19.0; SPSS, Tokyo, Japan).

## Results

### Executive function

**Fig. 2:**
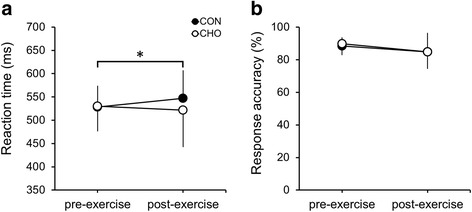
Mean reaction time (**a**) and mean response accuracy (**b**) in the incongruent Stroop Color and Word Test before and after exercise in CHO and CON. The black and white dots indicate the mean in CON and CHO, respectively. Data are expressed as means ± SD. (a) Reaction time effects, condition (*P* = 0.383), time (*P* = 0.594), interaction (*P* = 0.028). (b) Response accuracy effects, condition (*P* = 0.763), time (*P* = 0.194), interaction (*P* = 0.491). ^*^ indicates significant increase (*P* < 0.05) from pre-exercise to post-exercise in CON

The results of the reaction time and the response accuracy in the incongruent trials of the Stroop Color and Word Test are shown in Fig. 2. There was a significant interaction on the reaction time (*F*_[1, 7]_ = 7.625, *P* = 0.028, ${\eta}_p^2$ = 0.521) (Fig. 2a). A significant simple effect of time was found only for CON (*F*_[1, 7]_ = 7.501, *P* = 0.029, ${\eta}_p^2$ = 0.517), in which the reaction time significantly increased from pre-exercise to post-exercise. There were no main effects of condition (*F*_[1, 7]_ = 0.864, *P* = 0.383, ${\eta}_p^2$ = 0.110) and time (*F*_[1, 7]_ = 0.313, *P* = 0.594, ${\eta}_p^2$ = 0.043) on the reaction time. No significant main effects or interaction on the response accuracy were observed (*P* > 0.1) (Fig. 2b). The change in reaction time without a significant change in response accuracy between pre-exercise and post-exercise indicated that there was no speed–accuracy trade-off.

### Blood analysis

Table 1 shows the concentrations of blood hormones at pre-exercise and post-exercise. The plasma epinephrine concentration showed a significant interaction (*P* = 0.037) and main effects of time (*P* = 0.004) and condition (*P* = 0.036). Significant simple effects of time were found for both mouth-rinsing conditions (CON, *F*_[1, 6]_ = 14.592, *P* = 0.009, ${\eta}_p^2$ = 0.709, CHO, *F*_[1, 6]_ = 12.239, *P* = 0.013, ${\eta}_p^2$ = 0.671), in which the plasma epinephrine concentration significantly increased from pre-exercise to post-exercise. A significant simple effect of condition was found only for post-exercise (*F*_[1, 6]_ = 7.196, *P* = 0.036, ${\eta}_p^2$ = 0.545), in which the plasma epinephrine concentration was significantly higher in CON than in CHO. The plasma NE concentration indicated significant main effects of time (*P* = 0.001) and condition (*P* = 0.033), and tended to differ between mouth-rinsing conditions (condition × time interaction, *P* = 0.084). The plasma NE significantly increased after exercise. The plasma ACTH concentration indicated a significant main effect of time (*P* = 0.009), in which the plasma ACTH concentration significantly increased from pre-exercise to post-exercise. The plasma ACTH concentration showed no significant main effects of condition and no interaction (*P* > 0.1). No statistical significance was observed for blood glucose concentration (*P* > 0.1).

**Table 1:** Mean ± standard deviation of the levels of blood metabolites and hormones before and after exercise in the two mouth rinsing conditions, and the results of the two-way repeated measures ANOVA with conditions (CHO and CON) and time (Pre-exercise and Post-exercise) as factors

	CON		CHO		Main effect						Interaction		
					Condition			Time			Condition × Time		
	Pre-exercise	Post-exercise	Pre-exercise	Post-exercise	*F*_[1.6]_	*P*	${\eta}_p^2$	*F*_[1.6]_	*P*	${\eta}_p^2$	*F*_[1.6]_	*P*	${\eta}_p^2$
Blood glucose (mg/dl)	71.7 ± 9.0	86.7 ± 22.4	75.3 ± 12.4	84.1 ± 15.3	0.009	0.929	0.001	4.957	0.061	0.415	3.748	0.094	0.349
Plasma ACTH (pg/ml)	22.0 ± 11.5	102.0 ± 54.3	26.9 ± 11.4	73.8 ± 45.1	1.449	0.274	0.195	14.508	**0.009**	0.707	3.330	0.118	0.357
Plasma norepinephrine (ng/ml)	0.54 ± 0.24	1.69 ± 0.65	0.44 ± 0.18	1.11 ± 0.60	7.551	**0.033**	0.557	31.400	**0.001**	0.840	4.291	0.084	0.417
Plasma epinephrine (ng/ml)	0.02 ± 0.01	0.31 ± 0.20	0.02 ± 0.01	0.11 ± 0.06	7.233	**0.036**	0.547	19.704	**0.004**	0.767	7.130	**0.037**	0.543

Bold *p-*values: significant effects

*CON* Mouth rinsing with water, *CHO* Mouth rinsing with carbohydrate solution

### Perceptual assessments

Table 2 shows the RPE and the subjective perceptions at pre-exercise and post-exercise in CHO and CON. There was no significant interaction on RPE (*P* = 0.185); however, there were significant main effects of time (*P* < 0.001) and condition (*P* = 0.030). The RPE significantly increased after exercise and was significantly lower in CHO than in CON. Perceptual fatigue significantly increased after exercise (*P* < 0.001); however, there was no main effect of condition and no interaction (*P* > 0.1). In perceptual arousal, there were no main effects of time and condition, and no significant interaction (*P* > 0.1). In terms of perceptual pleasure, there was a significant main effect of time (*P* = 0.013); however, there was no main effect of condition and no interaction (*P* > 0.1).

**Table 2:** Mean ± standard deviation of subjective measurements before and after exercise in the two mouth rinsing conditions, and the results of the two-way repeated measures ANOVA with conditions (CHO and CON) and time (Pre-exercise and Post-exercise)

	CON		CHO		Main effect						Interaction		
					Condition			Time			Condition × Time		
	Pre-exercise	Post-exercise	Pre-exercise	Post-exercise	*F*_[1.7]_	*P*	${\eta}_p^2$	*F*_[1.7]_	*P*	${\eta}_p^2$	*F*_[1.7]_	*P*	${\eta}_p^2$
RPE	7 ± 2	18 ± 2	7 ± 1	16 ± 2	7.326	**0.030**	0.511	141.383	**<0.001**	0.953	2.156	0.185	0.235
Perceptual fatigue	5 ± 4	17 ± 3	4 ± 3	15 ± 3	1.126	0.324	0.139	59.313	**<0.001**	0.894	0.628	0.454	0.082
Perceptual arousal	14 ± 3	11 ± 4	11 ± 4	11 ± 3	1.493	0.261	0.176	1.299	0.292	0.157	2.333	0.170	0.250
Perceptual pleasure	13 ± 4	6 ± 5	11 ± 6	7 ± 4	0.189	0.677	0.026	11.124	**0.013**	0.614	0.771	0.409	0.099

Bold *p-*values: significant effects

*CON* Mouth rinsing with water, *CHO* Mouth rinsing with carbohydrate solution

## Discussion

The present study examined the effect of mouth rinsing with a carbohydrate solution on executive function after sustained moderately high-intensity exercise. The main result of the present study was that the reaction time significantly increased after exercise in CON but not in CHO, which resulted in a significant interaction (condition × time) on the reaction time in the incongruent Stroop test. This finding indicates that mouth rinsing with a carbohydrate solution attenuated the expected decline in executive function due to the sustained moderately high-intensity exercise, which supports our hypothesis.

An increased reaction time was observed after exercise in CON, but no such increase was observed in CHO. This result seems to indicate that mouth rinsing with the carbohydrate solution attenuated the exercise-induced decline in executive function. Mouth rinsing with glucose has been reported to improve the reaction time in incongruent trials in a resting condition [13]. Such a positive effect on the reaction time seems to have helped minimize the increase in reaction time after sustained moderately high-intensity exercise in CHO, preserving executive function.

The concentrations of plasma epinephrine and NE tended to be different between mouth-rinsing conditions, with a higher value in CON than in CHO after exercise. Such changes in stress hormones may be a possible reason for the positive effect of mouth rinsing with a carbohydrate solution on the decline in executive function. The Pearson correlation coefficient revealed a significant correlation in the changes between the plasma NE level and the reaction time in CON (*r* = 0.874, *P* = 0.010). The plasma epinephrine and NE directly affect synthesis and release of NE in the brain through the vagus nerve, and high levels of NE release are presumed to disrupt executive function [8, 33, 34]. The plasma NE level observed in the present study was comparable to the level reported in a previous study, which demonstrated that cognitive function was impaired after prolonged cycling at ~66% V̇O_2_max [10]. Therefore, executive function seems to be impaired by the exercise-induced increase in the epinephrine and NE levels in CON, and the preservation of executive function in CHO may be attributed, at least in part, to the inhibition of the excessive release of such stress hormones.

Mouth rinsing with a carbohydrate solution did not change the subjective perceptions of fatigue, arousal, and pleasure, indicating no significant interactions and no main effects of condition, although there were significant main effects of time. In a previous study, glucose intake enhanced executive function in comparison with the effects of an inert sucralose placebo, without differences in perceived fatigue and time availability, and in the number of perceived mistakes between conditions [22]. Therefore, the glucose-induced enhancement in executive function occurs separately from the subjective perceptions of fatigue, arousal, and pleasure during exercise.

The positive effect on executive function of mouth rinsing with a carbohydrate solution seems to be associated with a nonmetabolic pathway and to be unrelated to the glucose metabolic pathway. The blood glucose concentration showed neither significant interactions nor main effects of condition; however, a significant main effect of condition on the RPE was observed in this study. This indicates that the perceived exertion was reduced through a nonmetabolic pathway. A previous study demonstrated that mouth rinsing with a carbohydrate solution improved running performance because carbohydrate lowered the perception of effort at a given workload, as a result of activation of the reward center of the brain [35]. In support of this finding, it has been reported that the potential effect of mouth rinsing with carbohydrate is activation of reward-related brain regions, which include the anterior cingulate cortex and the striatum, and the dorsolateral prefrontal cortex [21]. The dorsolateral prefrontal cortex play a critical role during the incongruent condition of the Stroop test [36]. Thus, the positive effect of mouth rinsing with a carbohydrate solution on executive function could be particularly associated with reward-related brain regions. Furthermore, there is a possibility that the vagus nerve contributed to the preservation of executive function as another nonenergetic pathway [32]. Oral exposure to carbohydrate was found to increase heart rate variability, which was associated with prefrontal cortical activity through the vagus nerve, and persons with greater heart rate variability were observed to perform better on tasks related to the executive function [37]. Hence, the results of the present study suggest that non-energetic mechanisms such as the motivational reward pathways and the vagus nerve could possibly contribute to the preservation of executive function without increasing the metabolic energy level. Future studies are needed to determine whether nonmetabolic pathways would be associated with attenuation of the decline in executive function after sustained moderately high-intensity exercise.

One limitation of this study is its small sample size. A loss of significant interaction and main effects on some of the outcome measures, such as blood hormone concentrations and subjective perceptions, might have been due to the small sample size. However, the sample size of this study was determined from the preliminary results of the changes in the reaction time of the incongruent Stroop Color and Word Test after sustained moderately high-intensity exercise, which was the primary outcome measure of this study. On the basis of the means and variances of those data, seven participants were needed to detect changes in the reaction time to achieve a power of (1 – β) = 0.90 at *P* < 0.05. Thus, the sample size seems to be enough to detect changes in the reaction time. Four male and four female participants were included in the present study. Although some previous studies examined the effects of exercise or mouth rinsing with a carbohydrate solution on cognitive performance [12, 22, 38, 39] in the mixed gender samples, it remains unclear whether the effect of carbohydrate solution mouth rinsing on executive function in exercise varies according to gender. The performance of cognitive tasks could have been affected by the phases of menstrual cycle of the female subjects. However, the experiment was conducted during the follicular phase of their menstrual cycles; thus, the influence of the menstrual cycle, if at all, should be minimal. Further studies would be needed to elucidate gender differences in the effect of the carbohydrate solution mouth rinsing on executive function since hormonal responses could be different between males and females.

## Conclusions

Mouth rinsing with a carbohydrate solution attenuated a decline in executive function, which was induced by sustained moderately high-intensity exercise. Such attenuation of the decline in executive function seems to be unrelated to the metabolic pathway of carbohydrate. The findings of this study would be useful in designing interventions for preserving executive function in dynamic situations that require sustained moderately high-intensity exercise such as sports activities.
